# 

**Published:** 1888-05-26

**Authors:** 


### American Jottings.

We see that a St. Louis firm announces a new antiseptic called "Listerine." Sir Joseph ought to feel flattered, but has not carbolic acid the right *par excellence* to the title of "Listerine"?

"A Rambler," writing in the American *National Baptist*, comments very severely on the habit of promiscuous almsgiving, and quotes an instance of true charity shown without the expenditure of a penny. A lady was appealed to aid a poor family. She gave them no money, but investigated their condition. Her first effort was to put them in the way of making the most of their own resources. They were living in a cellar, damp, unhealthy, dark, supplied with few or no conveniences. She said to them, "Now, for less than you are paying here, you can get a tenement above the ground, better in all respects." But they did not know where to look for it. She took time, and looked it up. Then she said, "What are you earning, and what are the older children earning?" She put them in the way of making a slight increase in income. She insisted on their putting something in the savings bank. Of course, there were trials of temper and wearings of patience. It took time, strength, nerves. It would have been vastly easier to have given the family a dollar, five dollars, ten dollars, and let that be the end of it, and let them sink deeper and deeper into the slough of pauperism. But her course, though she did not give a penny in money, was the salvation of the family.

The *Monthly Register* of the Philadelphia Society for Organising Charity strongly advocates the giving of loans to working men in order to enable them to tide over seasons of difficulty. At present, if forced by sickness or any other cause into idleness, they can obtain loans only at rates so extortionate as to be absolutely ruinous. It would be a true charity—more really helpful than gifts of the same sum—for those who are interested in the poor to lend them money at such times, and—this is equally important—see that it is repaid when the crisis is over.

The Essex County Medical Society, New Jersey, has begun a war upon physicians practising without a proper diploma, and the Committee on Illicit Practice has been directed to secure the indictment by the grand jury of three persons who have, it is alleged, no legal status as physicians.

Dr. William Benjamin Goldsmith, Superintendent of the Butler Hospital for the Insane, at Providence, Rhode Island, died on March 21st of acute pneumonia, aged 34. A resolution, expressing deep regret at his loss and sympathy with his family, was passed by the New England Psychological Society on hearing of the event.

Dr. F. Semeleder, of Mexico, reports the following curious case of congenital baldness: "A girl of twelve was brought to me; when she was born she had only a few long hairs; soon they fell out, and no hair has ever grown on her head. The head has not the shiny appearance of that of a bald man, but the skin is dry, and looks like shagreen leather. The girl has no other deformity. She has brothers and sisters, none of whom are bald, nor are her parents."

Dr. Gouverneur M. Smith, of New York, has an article in the *Medical Record* of last month, entitled "Wasted Sunbeams—Unused House-tops," in which he suggests that "architectural ingenuity, coached by sanitary science" should contrive somemethod of using the acres of house-tops a city affords for out-door recreation and protection from invalidism. There is a "solarium" at the New York Hospital, which is said to be no mean aid to convalescence, and there can be no doubt that the sun baths of the ancient Romans had a distinct sanitary value. Here in London our roofs are not as a rule adapted for exercise of other than a severely gymnastic kind, but alterations might be introduced, so that most houses could have something corresponding to the Italian *loggia*. Even then the "blacks" would have to be reckoned with; but they would wash off while the good effect of the sunbeams would remain.

### Special Notice to Newsagents, Advertisers, and the Trade.

### CHANGE OF OFFICE AND PUBLISHER.

NOTICE is hereby given that the Office of THE HOSPITAL will henceforth be at 38, Old Jewry (Cheapside end), E.C., to which address all communications should be sent. Mr. A. Doig has been appointed Advertising Agent, and Mr. J. H. S. Hanning, Manager. All Cheques and Post Office Orders should be made payable to J. H. S. Hanning, 38, Old Jewry, E.C. crossed Lloyds, Barnetts, and Bosanquets Bank (Limited).

### To Secretaries and Matrons.

THE Editor will be glad to have a copy of the Regulations, and also of every Report issued from the Press of Asylums, Hospitals, Convalescent and Nursing Institutions, as well as those of other Charities, so soon as they are published, and an early copy in duplicate of all appeals for funds. Notices of Annual and other Meetings, Festival Dinners, etc., will be welcomed. All such communications should be addressed to *The Editor, The Lodge, Porchester Square, London, W.* Any superintendent, secretary, matron, or other chief official who will regularly send such documents and information will be registered to receive free of cost a cover for binding THE HOSPITAL in half-yearly volumes on sending name and address to the publisher.

I had rather feel compunction than understand the definition thereof.—*Thomas à Kempis.*

### AMUSEMENTS WITH PROFIT FOR ALL AGES.

**Rules.**—All answers must be addressed to the PRIZE EDITOR, THE HOSPITAL, 38, Old Jewry, Cheapside, London, E.C., not later than the *first* post on Thursday next. The full name and address must in all cases be given, but a *nom de plume* may be added for publication. Only one side of the paper must be written on.

### FOR MEN AND WOMEN.

**Rule.**—Competitors can enter for *all* quarterly competitions, but no competitor may take more than *two quarterly prizes* during the year.

### First Quarterly Poetical Competition.

Commences May 26th, 1888. Ends August 18th, 1888.

This quarter a PRIZE of ONE GUINEA will be given to the competitor who gains the highest number of marks for original poems, on any subjects suitable for insertion in this paper. All contributions sent in will be credited according to merit, eighteen marks being the highest score for any one contribution. This competition to alternate weekly with the set acrostic competition.

### Acrostic Competition.

This quarter the original acrostic competition will be discontinued, and A PRIZE of HALF-A-GUINEA will be given to the competitor who gains the highest score for solution of acrostics set. *One mark* only will be given to the competitors who solve all the lights of each acrostic.

Exception will be made in the case of *quotation acrostics*, when *two marks* will be given, *one* for the correct solution of all *lights*, and *one* mark for the *source* of the quotations.

This week credit will be given for

**No 1 Original Poem.**

**Results of No. 6 Original Acrostic.**

Brownie (10), Patience (10), A. M. E. (10).

### THE WORD COMPETITION.

Third Quarterly Competition commenced

May 5th, 1888; ends July 28th, 1888.

Three prizes of one guinea, 15*s.* and 10*s.* 6*d.* will be given each quarter to the three competitors who obtain the highest number of marks by making the largest number of words derived from the word or phrase set for anatomical dissection; that for this, the *fourth* week of the quarter, being

"OAK APPLE."

OBSERVE.—Proper names, abbreviations, foreign words, words of less than four letters, and repetitions are barred; plurals, and past and present participles, of verbs are allowed. Nuttall's *Standard* dictionary only to be used.

N.B.—All words sent in must be arranged *alphabetically*, with correct total affixed.

**Results of Second Week.**

*Rare Bits.*—Patience (143), Tinie (141), Lightowlers (130), Lauriston (127), Dollie (124), Sym (124), Carmen (114), Reldas (113), Brisbane (109), Dodo (106), Puff (101), Geranium (83), Robe (76), Ione (72)

*Dodo* should have been credited with (29) marks last week for above competition.

### THE CHILDREN'S CORNER.

PRIZES.

FOR MOST CORRECT ANSWERS TO PUZZLES.

This Commenced October 1st, 1887.

One Pound a Month.

A PRIZE OF THREE GUINEAS

to the Competitor who shall have sent in the largest number of correct or best answers during the twelve months.

**Puzzles—Fourth Week.**

No. 13. GEOGRAPHICAL ACROSTIC.—1. The city of a hundred gates. 2. To hold without right. 3. A city of Italy, the birthplace of Ariosto. 4. A fortified city of European Russia. 5. An island to the west of Scotland. 6. A river of France. Initials will give the name of a country in Europe, and the finals something for which it is celebrated.

No. 14. Who can explain the following picture puzzle?

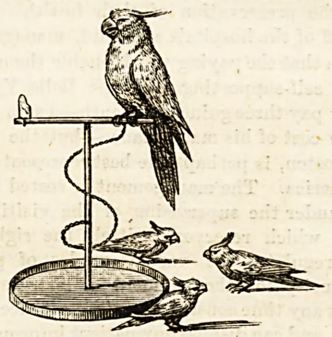

No. 15 BIOGRAPHICAL CHARADE.—My *first* is a nut resembling a filbert; my *second* is used by most fishermen; and my *whole* the name of a monk, who first invented glass in England.

No. 16. ENIGMA.

It came though I fetched it, when come it was gone,
It stayed but a moment, it could not stay long;
I ask not who saw it—it could not be seen,
And yet might be felt by the king or the queen.

**Results of Second Week Puzzles.**

No. 5. CHARADE.—Coal-mine = Coalmine.

No. 6. DOUBLE ACROSTIC.

P ——— S

H ayt I

I relan D

L ittle Joh N

I r E

P air Y

No. 7. ARITHMETICAL PUZZLE.—The total number of pears was 15.

No. 8. TRANSPOSITION.—Pan, pen, pin, pun.

*All right.*—Mount Edgecumbe, A. C. K., Geranium, G. H., Alsie, R. H., McCulloch, Nunkey, Spider, Hercules. *Three right.*—Pickles, Jumbo, Gem, Penelope, Scrooge, Plummy Fluff

**Notices to Correspondents.**

*The Young Canadian.*—Your answers received, but cannot be credited, as the postage was unpaid.

